# The Staple Crown

**Published:** 1903-11-15

**Authors:** F. L. Marshall

**Affiliations:** Boston, Mass.


### THE STAPLE CROWN.

BY DR. F. L. MARSHALL, OF BOSTON, MASS.

The principal difference between Dr. Carmichael's crown and the author's is that he (Dr. Carmichael) burnishes gold into grooves made in the teeth to be crowned, while Dr. Marshall uses a platino-iridium staple. This staple crown he has used for different purposes for five years or more, with very gratifying results.

As to the preparation of any of the six front teeth for the crown, the author says: "Take the enamel fissure bur, have your assistant keep a fine stream of cold water running over the tooth, and quickly cut into the side of the tooth to the depth of the bur. Repeat the same on the opposite side, having the grooves come just back of the contour. Then connect the grooves across the tooth by the use of an enamel bur or a small stone. If the articulation is very close, grind off enough to allow for the thickness of the crown. Then knife-edge your tooth from the cross groove to the point as you would an artificial tooth before backing up.

"Next select your piece of platino-iridium wire, a trifle smaller than the bur; bend a right angle on the end long enough to fit one proximal groove. Take the distance across between the grooves in your eye, and bend another right angle in the wire at that distance, thus forming the 'staple' which gives the crown its name. Cut the ends of the staple the proper length to fit the grooves, having the cross-bar flush with the palatal portion of the tooth. Now take a thin piece of pure gold, from 30 to 35 gauge, large enough to fully cover the back ot the tooth. Take your staple in a pair of pliers, and hold in contact with the piece of pure gold at a slight angle. Solder with 22-karat solder at the point of contact. Bend the gold loosely around the sides of the staple, and trim cervical portion. Place the staple into the grooves, and burnish the gold firmly to one side. Remove and solder. Replace the crown, and burnish all around back of tooth tightly to the opposite side. Remove and solder. Now replace and burnish all around the free edges. Trim off the surplus; remove; flow 20-karat solder over the outside of the crown, taking particular pains to leave or to keep the edges of the pure gold to about one-thirty-second of an inch free from solder These edges are to be burnished tightly to the tooth when the crown is cemented into place. The burnishing is to be done before the cement hardens.

"The construction of a bicuspid or molar crown is very different from one for any of the six front teeth. The first thing to do is to grind off the inner cusp and straighten the walls of the back part of the tooth; then cut the grooves in the anterior cusp just as you would in a cuspid. Cut a band of 22-karat gold as you would to make a full crown only not quite so long. Solder one side of the staple about one-sixteenth of an inch from one end of the band. Place the staple on the tooth. Draw the band around the tooth to the opposite side. Burnish tightly to the neck. Remove; push the free end of the staple back about the distance of one line, so as to assure a tight fit, and then solder. Strike up cusps, preferably of 22-karat gold, fit to the top of the band, and solder at the inner point of contact. Put in place on the tooth. Allow the patient to bite firmly to give correct occlusion. Burnish to the cutting edge of outer cusp. Remove; trim and fill the inner cusp with solder up to the cross-bar of the staple. Allow the solder to unite the joints between the cusps, and the band at the outer or buccal cusp. Burnish all around the edges, and the crown is finished.

"The lower teeth are treated in the same way as the upper, only that a smaller wire must be used."—*Review.*

